# The Development of the Skull of the Egyptian Cobra *Naja h*. *haje* (Squamata: Serpentes: Elapidae)

**DOI:** 10.1371/journal.pone.0122185

**Published:** 2015-04-10

**Authors:** Eraqi R. Khannoon, Susan E. Evans

**Affiliations:** 1 Zoology Department, Faculty of Science, Fayoum University, Fayoum, 63514, Egypt; 2 Department of Cell and Developmental Biology, University College London, Gower Street, London, WC1E 6BT, England, United Kingdom; University of Texas Southwestern Medical Center, UNITED STATES

## Abstract

**Background:**

The study of craniofacial development is important in understanding the ontogenetic processes behind morphological diversity. A complete morphological description of the embryonic skull development of the Egyptian cobra, *Naja h*. *haje*, is lacking and there has been little comparative discussion of skull development either among elapid snakes or between them and other snakes.

**Methodology/Principal Findings:**

We present a description of skull development through a full sequence of developmental stages of the Egyptian cobra, and compare it to other snakes. Associated soft tissues of the head are noted where relevant. The first visible ossification centres are in the supratemporal, prearticular and surangular, with slight ossification visible in parts of the maxilla, prefrontal, and dentary. Epiotic centres of ossification are present in the supraoccipital, and the body of the supraoccipital forms from the tectum posterior not the tectum synoticum. The venom glands are visible as distinct bodies as early at stage 5 and enlarge later to extend from the otic capsule to the maxilla level with the anterior margin of the eye. The gland becomes more prominent shortly before hatching, concomitant with the development of the fangs. The tongue shows incipient forking at stage 5, and becomes fully bifid at stage 6.

**Conclusions/Significance:**

We present the first detailed staging series of cranial development for the Egyptian cobra, *Naja h*. *haje*. This is one of the first studies since the classical works of G. de Beer and W. Parker that provides a detailed description of cranial development in an advanced snake species. It allows us to correct errors and misinterpretations in previous accounts which were based on a small sample of specimens of uncertain age. Our results highlight potentially significant variation in supraoccipital formation among squamates and the need for further research in this area.

## Introduction

Snake skulls show considerable phylogenetic and functional variation across the clade (Serpentes) [1,2]. The cranial anatomy of adult snakes has recently been reviewed in detail [3], but there has been limited comparative work on cranial development in snake embryos. Much of the existing literature is based either on serial sections [4,5] or focuses on the description of a few representative stages [6,7]. Cleared and stained embryonic series, such as those described for the Monocled Cobra, *Naja kaouthia*, [8], the African Rock Python, *Python sebae* [9], and the lamprophiid *Boaedon fuliginosus* [10], can provide a more comprehensive basis for comparison, but are relatively rare. Computed microtomography offers a new approach, as for the viperid *Bothropoides jararaca* [11], although standard scanners do not have the resolution needed to image mesenchymal condensations or chondrocranial cartilages.

Elapids form a geographically widespread group of venomous snakes that includes kraits, coral snakes, mambas, sea snakes and cobras. They have a proteroglyphous dentition characterised by the possession of hollow, fixed, front-fangs through which venom is injected from large, supralabial venom glands. Of the sixty one recognised genera of elapids, cobras are probably the most familiar to the non-specialist due to the extended 'hood' that typically forms part of the threat display. True cobras (genus *Naja*) occur throughout Africa, Asia and the Middle East. The Egyptian cobra, *Naja h*. *haje*, is a large, slender snake that is found in a wide range of habitats in the Nile Valley, western Egyptian desert, and the Mediterranean coastal desert. As a relatively common Egyptian snake, various aspects of its biology have been comparatively well studied. In a series of detailed papers, Kamal and Hammouda [7] and Kamal et al. [12–14] described the embryonic skull of *N*. *h*. *haje*. However, the embryos were extracted from eggs recovered from a single naturally occurring nest. As a result, very few embryos were available and the date of oviposition was not known. This makes it difficult to compare the development of the skull with that of other snakes.

Until 2013, a detailed developmental series had only been described for one cobra species, the Asian Monocled Cobra, *N*. *kaouthia* [8]. We recently supplemented that study with a comparative staging table for *Naje h*. *haje*, based on a large sample of embryos [15]. Allowing for differences in incubation temperature, we found only relatively minor differences in the timing of developmental stages, based on external features, between *N*. *kaouthia* and *N*. *h*. *haje* [15]. However, comparison of skeletal development was limited by the problems with embryo age and sample size in the work of Kamal and colleagues [7,12–14]. We therefore cleared and stained a staged subsample from our *N*. *h*. *haje* series in order to examine skull development, focusing particularly on the early stages that were not covered by the work of Kamal and colleagues. This series, described below, allows a fuller comparison between *N*. *kaouthia* and *N*. *h*. *haje*, and provides a more detailed basis for comparison with other snakes. In addition, it permits a re-assessment of some points of controversy in relation to snake skull development, such as the formation of the supraoccipital bone.

## Materials and Methods

Eggs were collected from wild caught gravid female cobras (*Naje h*. *haje*) kept in cages under field conditions. Fertilised eggs were removed at oviposition, and transferred to the laboratory where they were placed in ventilated containers filled with perlite (at 85–90% moisture) and incubated at a constant temperature of 30°C. Under these conditions, hatching usually occurred after 51–54 days.

Eggs were opened each day post-oviposition (dpo). The extracted embryos were placed and examined in Petri dishes filled with phosphate buffered saline (PBS), and were then fixed in 10% buffered formalin.

**Fig 1:**
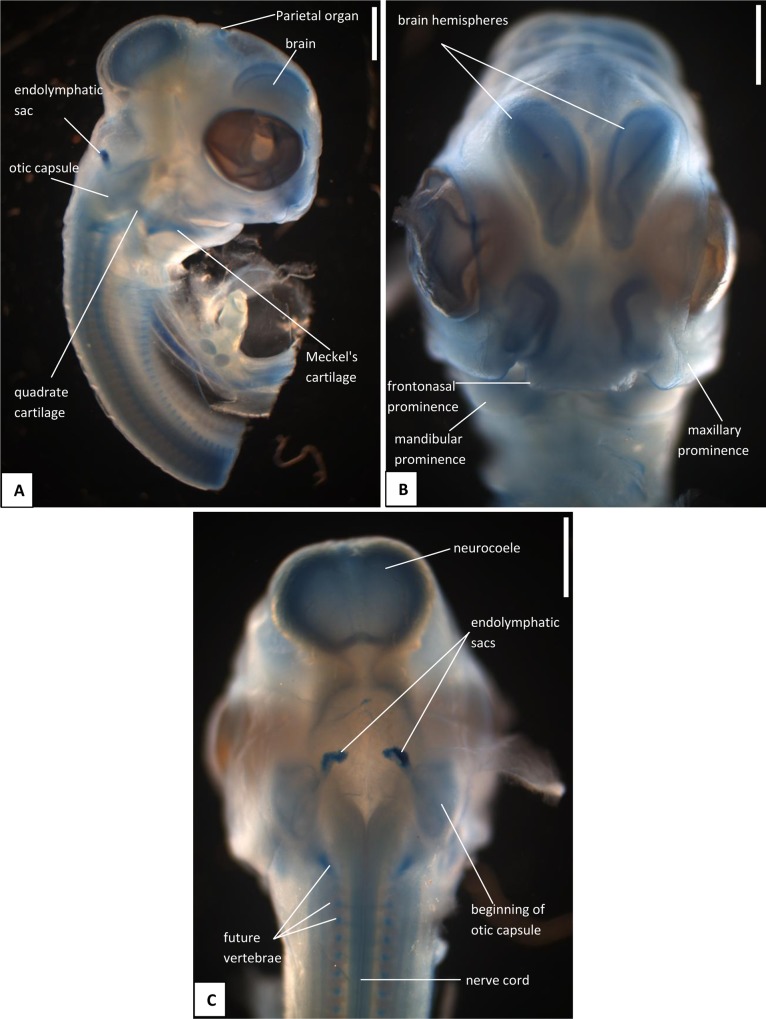
Stage 4 (13 dpo), craniofacial development of *Naja h*. *haje*. A) right lateral view; B) anterior view; C) dorsal view. Scale bars: 1mm.

**Fig 2:**
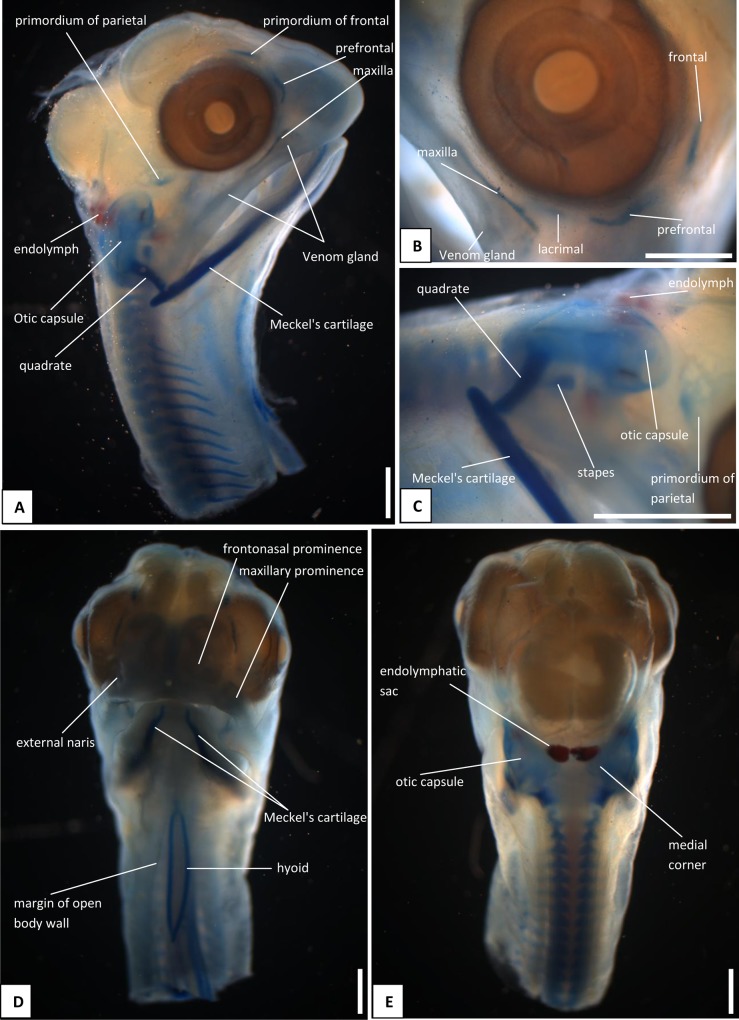
Stage 5 (17 dpo), craniofacial development of *Naja h*. *haje*. A) right lateral view; B) detail of circumorbital region; C) detail of quadrate and jaw joint; D) anterior view; E) dorsal view. Scale bars: 1mm.

**Fig 3:**
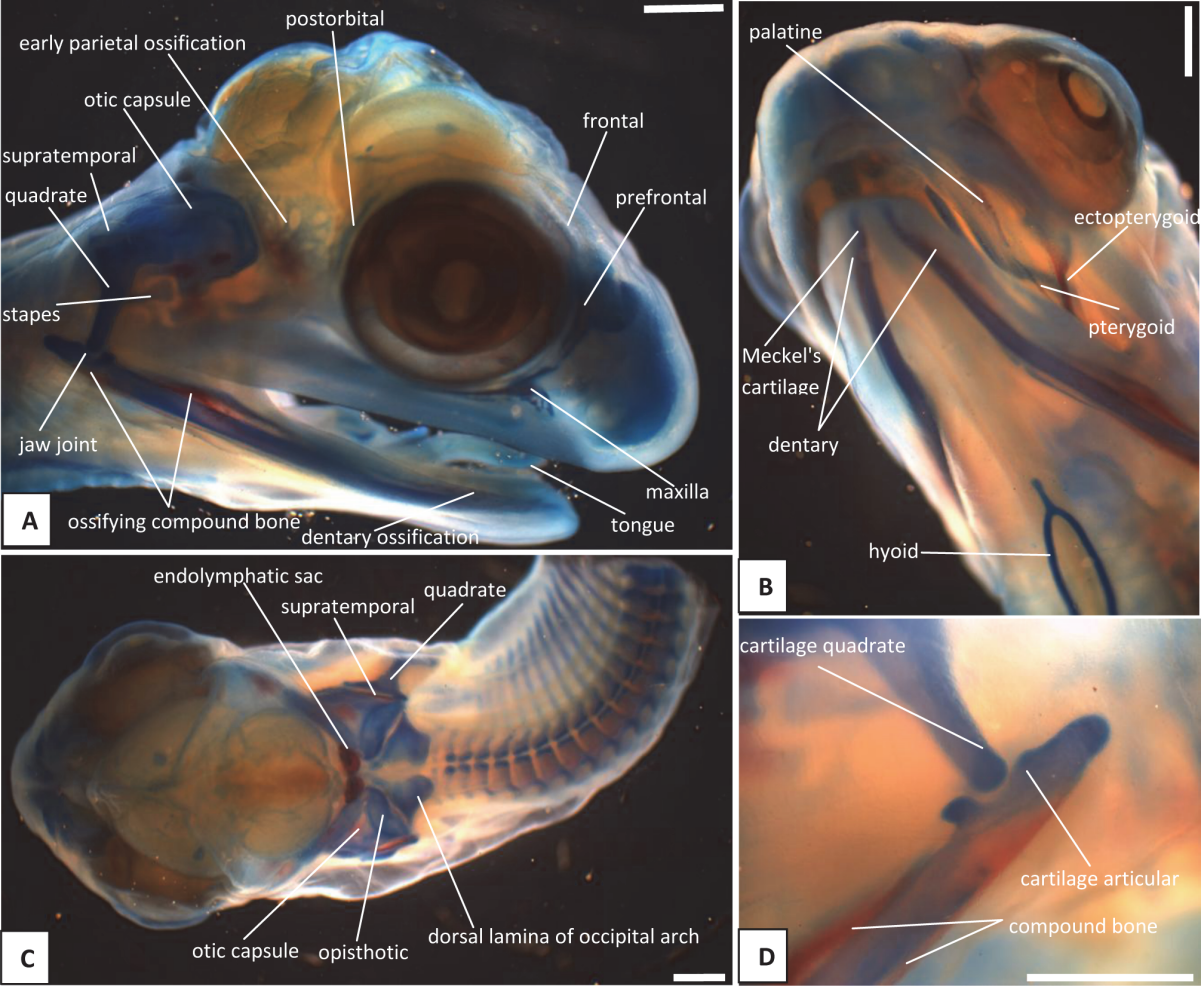
Stage 6a (22 dpo), craniofacial development of *Naja h*. *haje*. A) right lateral view; B) oblique left ventrolateral view; C) dorsal view; D) detail of quadrate and jaw joint. Scale bars: 1mm.

**Fig 4:**
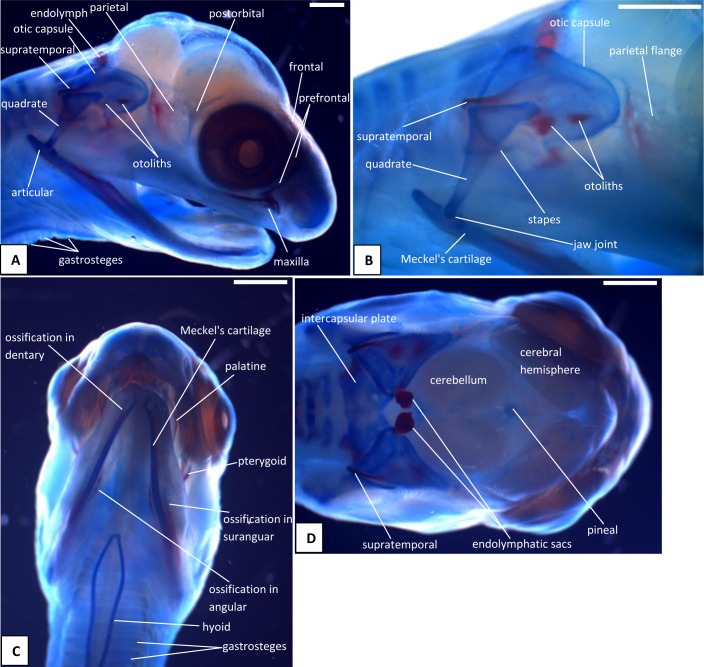
Stage 6b (24 dpo), craniofacial development of *Naja h*. *haje*. A) right lateral view; B) detail of right quadrate and otic capsule; C) ventral view; D) dorsal view. Scale bars: 1mm.

**Fig 5:**
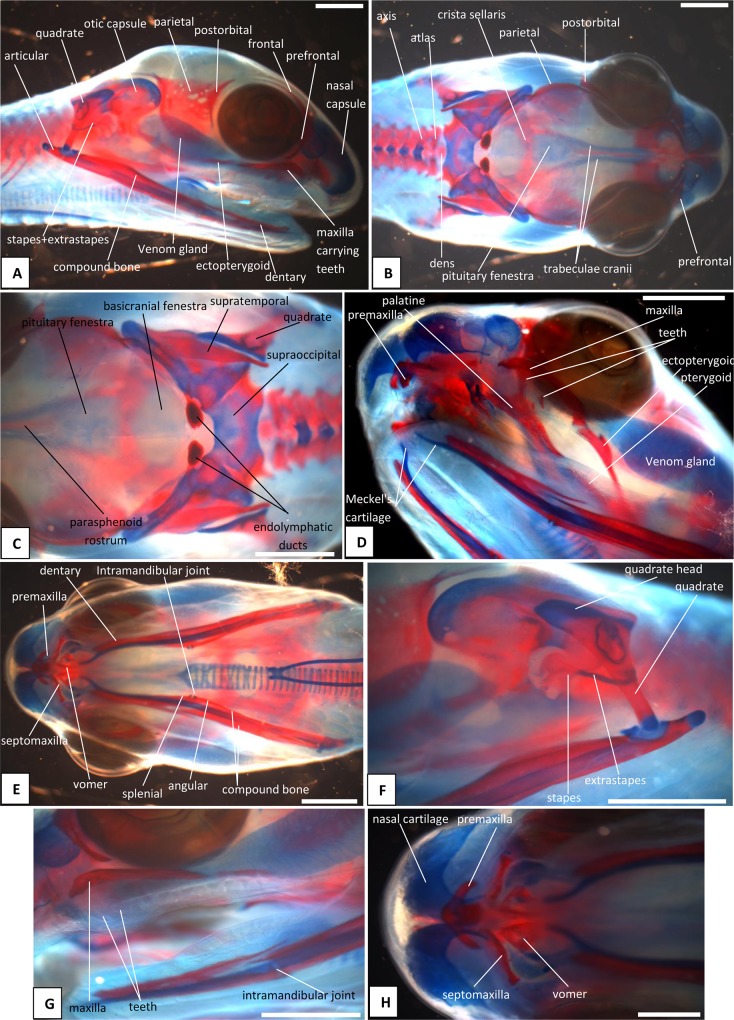
Stage 7a (33 dpo), craniofacial development of *Naja h*. *haje*. A) right lateral view; B) dorsal view; C) detail of occipital region; D) left ventrolateral view; E) ventral view; F) detail of left otic capsule and quadrate; G) detail of maxilla and mandibles; H) detail of anteroventral region. Scale bars: 1mm.

**Fig 6:**
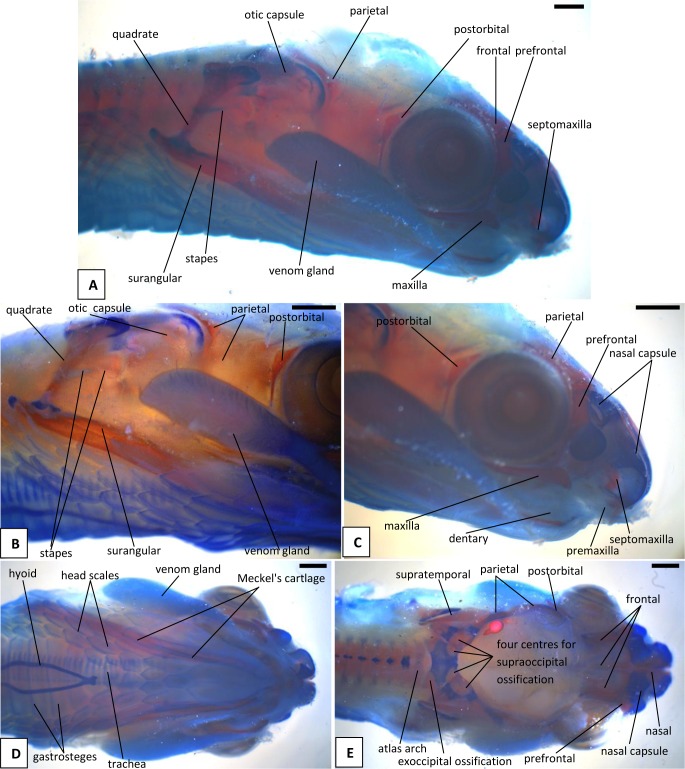
Stage 7b (38 dpo), craniofacial development of *Naja h*. *haje*. A) right lateral view; B) detail of right otic capsule and quadrate; C) detail of right rostrum and orbit; D) ventral view; E) dorsal view. Scale bars: 1mm.

**Fig 7:**
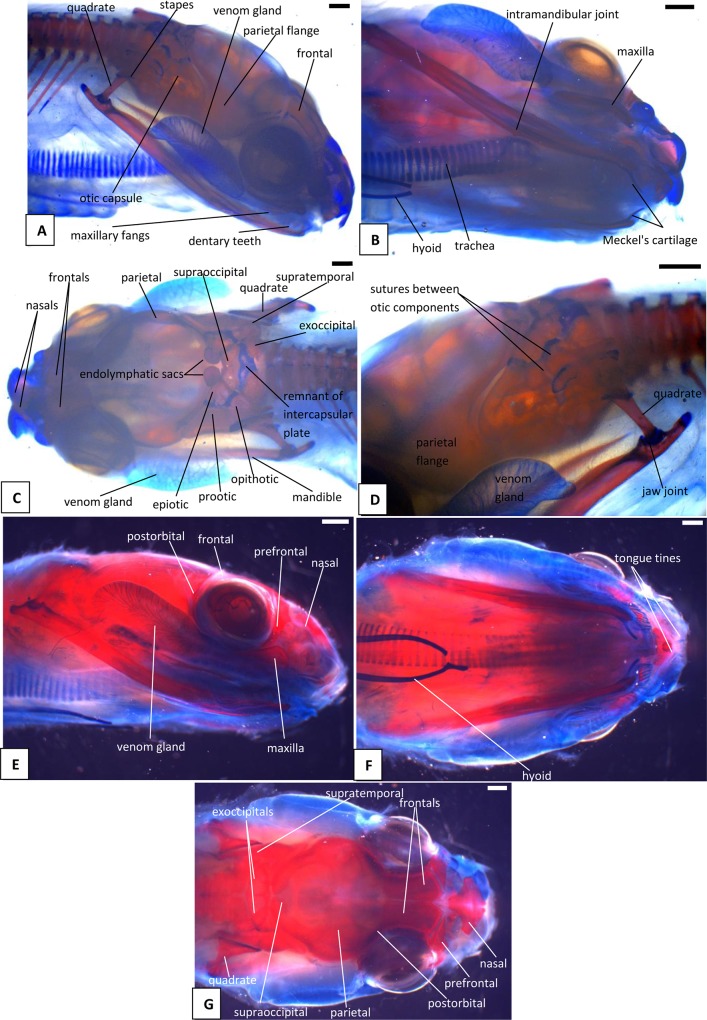
Stage 8 (42 dpo) (A-D) and Stage 9 (47dpo) (E-G), craniofacial development of *Naja h*. *haje*. A) right lateral view (42 dpo); B) right ventrolateral view (42 dpo); C) dorsal view (42 dpo); D) detail of left jaw joint and otic capsule (42 dpo); E) right lateral view (47 dpo); F) ventral view (47 dpo); G) dorsal view (47 dpo). Scale bars: 1mm.

**Fig 8:**
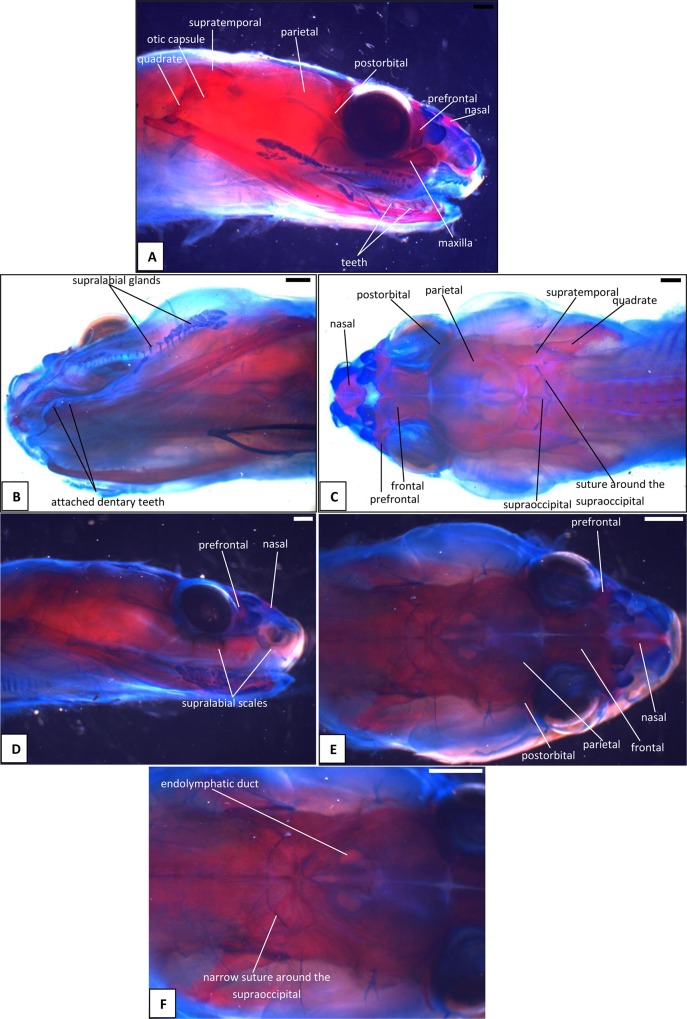
Stage 10a, b (53 dpo) (A-C) and Stage 10c (pre-hatchling) (D-F), craniofacial development of *Naja h*. *haje*. A) right lateral view (53 dpo); B) left ventrolateral view (53 dpo); C) dorsal view (53 dpo); D) right lateral view (pre-hatchling); E) dorsal view (pre-hatchling); F) detail of occipital region (pre-hatchling). Scale bars: 1mm.

The heads of snake embryos used in the current study were from samples previously preserved in 10% buffered formalin and used in our previous study [15]. For the skull series, we took embryos at 13 dpo (Fig 1), 17 dpo (Fig 2), 22 dpo (Fig 3), 24 dpo (Fig 4), 33 dpo (Fig 5), 38 dpo (Fig 6), 42 dpo (Fig 7A–7D), 47 dpo (Fig 7E–7G), 51 dpo, 53 dpo (Fig 8A–8C), and immediately pre-hatchling (~54 dpo, Fig 8D–8F). Based on our previous staging series [15], these correspond to stages 4, 5, 6a, 6b, 7a, 7b, 8, 9, 10a, 10b, 10c respectively. In the descriptions that follow, we use a double indicator, stage:dpo, with a, b, c used in longer stages to indicate early, middle, or late. By comparison with our specimens, we estimate the principal embryonic specimen described by Kamal et al. [12] to be at stage nine and equivalent to our 9a:47dpo embryo. Note also that we have retained the term embryo for the developing snake up to hatching. In humans, embryo is applied only to the early stages of development during which the organs and other adult structures are forming. Fetus is then used for the longer maturation stage up until birth. Some authors (e.g. [16]), recommend the same usage for reptiles and birds, but this is practice is not widespread.

In order to facilitate the study of skull development, the embryos were cleared and stained using a basic ethanol-KOH-Glycerol Alizarin red-Alcian blue staining protocol modified from Hanken and Wassersug [17]. The heads from embryos at different stages were post-fixed in 70% ethanol. The samples were then dehydrated through 80% and 90%, 1–2 hours at each step, and transferred to 95% for seven days. The samples were then placed in acetone for three days, before immersion in the staining solution (alcian blue+ alizarin red+ glacial acetic acid+ 70% ethanol) for three days. The samples were taken through different washes of distilled water for 3 hours. They were then transferred to 1% KOH and taken through different ratios of glycerol:KOH, 1:4, 1:3, 1:1. The stained heads were stored in glycerol.

Images were captured using an Olympus SZH10 stereo microscope with a Rebiga 2000R camera attachment, and using QCapture Pro at 1200x1600 resolution.

In the descriptions that follow, we have used the presence of red, alizarin, staining to indicate the beginning of ossification. We accept that this may underestimate the timing of onset [11], but it provides the most reliable indicator in the absence of histological examination of all tissues.

### Ethics statement

The embryo heads were from preserved snake embryos used previously [15] and were decapitated before fixation, and all efforts were made to minimize suffering. The entire animal work of that published paper and the current study was approved and permitted by the committee of Zoology Department, Fayoum University, Faculty of Science on the Ethics of Animal Experiments.

## Results

Tables 1 and 2 summarise the main stages in the development of the skull bones of *N*. *h*. *haje*, and compares this to other snakes that have been examined in a similar way. Rather than describe the development stage by stage (as figured), we have taken a regional approach.

**Table 1:** Summary of a comparison between craniofacial development of *Naja h*. *haje* (this study) and *N*. *kaouthia* [8], *Python sebae* [9]: DPO, days post-oviposition; DG, days of growth.

		***Naja h*. *haje***		***N*. *kaouthia*** [8]		***Python sebae*** [9]	
	**Developmental events**	**whole mount**	**% devtime**		**% devtime**		**% devtime**
**1**	Vertebral centra ossification	5: 17 DPO	31%	3:DPO 9–15	14–25%		
**2**	Prefrontal ossification	6: 22 DPO	41%	8: DPO28-38	43–63%	4: 18 DPO	20–23%
**3**	Maxillary ossification	6: 22 DPO	41%	<7: DPO 24–28	37–48%	4: 18 DPO	20–23%
**4**	Supratemporal ossification	6: 22 DPO	41%	<7: DPO 24–48	37–48%	4: 18 DPO	20–23%
**5**	Compound bone	6: 22 DPO	41%	8: DPO28-38	43–63%	4: 18 DPO	20–23%
**6**	Dentary ossification	6: 22 DPO	41%	3:DPO 9–15	14–25%	4: 18 DPO	20–23%
**7**	Ectopterygoid ossification	6: 22 DPO	41%	3:DPO 9–15	14–25%	6: 33 DPO	37–41%
**8**	Palatine ossification	6: 22 DPO	41%	3:DPO 9–15	14–25%	4: 18 DPO	20–23%
**9**	Pterygoid ossification	6: 22 DPO	41%	3:DPO 9–15	14–25%	3: 11–12 DPO	12–15%
**10**	Neural arches	6: 22 DPO	41%	8: DPO28-38	43–63%		
**11**	Rib ossification	6: 22 DPO	41%	8: DPO28-38	43–63%		
**12**	Basioccipital ossification	6: 22 DPO	41%	8: DPO28-38	43–63%	8: 54 DPO	60–68%60%
**13**	Otic elements (prootic) begin	6: 22 DPO	41%	8: DPO28-38	43–63%	7. 44 DPO	49–55%
**14**	Exoccipital ossification	6b: 24 DPO	44%	8: DPO28-38	43–63%	6: 33 DPO	37–41%
**15**	Quadrate ossification	6b: 24 DPO	44%	8: DPO28-38	43–63%	7: 44 DPO	49–55%
**16**	Premaxilla ossification	6b: 24 DPO	44%	3:DPO 9–15	14–25%	6: 33 DPO	37–41%
**17**	Vomer ossification	6b: 24 DPO	44%	8: DPO28-38	43–63%	6: 33 DPO	37–41%
**18**	Frontal ossification	7: 33 DPO	61%	8: DPO28-38	43–63%	6: 33 DPO	37–41%
**19**	Parietal ossification	7: 33 DPO	61%	8: DPO28-38	43–63%	6: 33 DPO	37–41%
**20**	Postorbital ossification	7: 33 DPO	61%	8: DPO28-38	43–63%	6: 33 DPO	37–41%
**21**	Stapes ossification	7: 33 DPO	61%				
**22**	Nasal ossification	7: 33 DPO	61%	8: DPO28-38	43–63%	4: 18 DPO	20–23%
**23**	Teeth on maxilla	7: 33 DPO	61%	8: DPO28-38	43–63%	7: 44 DPO	49–55%
**24**	Angular ossification	7: 33 DPO	61%	8: DPO28-38	43–63%	6: 33 DPO	37–41%
**25**	Splenial ossification	7: 33 DPO	61%	8: DPO28-38	43–63%	4: 18 DPO	20–23%
**26**	Articular ossification	7: 33 DPO	61%	3:DPO 9–15	14–25%	6: 33 DPO	37–41%
**27**	Septomaxilla ossification	7: 33 DPO	61%			6: 33 DPO	37–41%
**28**	Parasphenoid rostrum ossification	7: 33 DPO	61%	10: DPO51+	78–85%	7: 44 DPO	49–55%
**29**	Basisphenoid ossification	7: 33 DPO	61%	8: DPO28-38	43–63%	7: 44 DPO	49–55%
**30**	Supraoccipital ossification	7: 33 DPO	61%	8: DPO28-38	43–63%	?6:33DPO	37–41%
**31**	Teeth on dentary	7b: 38 DPO	70%	8: DPO28-38	43–63%	10: 75 DPO	83–94%
**32**	Palatal teeth	8: 42 DPO	78%	10: DPO51+	78–85%	10: 75DPO	83–94%
**33**	Laterosphenoid						
**34**	Hatching days DPO	51–54 DPO		60–65 DPO		80–90 DPO	

**Table 2:** Continued comparison between craniofacial development of *Naja h*. *haje* (this study) and *Elaphe obsoleta* [29], *Acrochordus granulatus* [18], *Boaedon fulinginosus* [10], *Nerodia taxispilota* [30], *Psammophis sibilans* [7], and *Natrix tesselata* [31], *Bothropoides jararaca* [11].

	***Elaphe obsoleta*** [29]		***Boaedon fulinginosus*** [10]		***Nerodia taxispilota***[30]	***Psammophis sibilans*** [7]	***Natrix tesselata*** [31]	***Bothropoides jararaca*** [11]	***Acrochordus granulatus*** [18]
		**% devtime**		**% devtime**					
**1**			5: 14–16 DPO	22–29%	10: 25 DG				
**2**	?: 36 DPO	56–59%	8: 24–29 DPO	37–52%	15: 33 DG	35b DPO	3: DPO 21	SES 3	1or2
**3**	?: 30 DPO	47–49%	8: 24–29 DPO	37–52%	16: 34 DG	35a DPO	2: DPO 13	SES 2	1
**4**	?: 36 DPO	56–59%	5: 14–16 DPO	22–29%	15: 33 DG	35b DPO		SES 3	1
**5**	?: 30 DPO	47–49%	5: 14–16 DPO	22–29%	13: 28 DG	35b DPO		SES 2	1
**6**	?: 41 DPO	63–67%	5: 14–16 DPO	22–29%	14: 29 DG	35b DPO	1: DPO1-2	SES 3	1
**7**	?: 24 DPO	38–39%	8: 24–29 DPO	37–52%	16: 34 DG	35b DPO	1: DPO1-2	SES 2	1
**8**	?: 24 DPO	38–39%			12: 27 DG	35a DPO	1: DPO1-2	SES 2	1
**9**	?: 24 DPO	38–39%	8: 24–29 DPO	37–52%	12: 27 DG	35a DPO	1: DPO1-2	SES 2	1
**10**			8: 24–29 DPO	37–52%	13: 28 DG				
**11**			8: 24–29 DPO	37–52%	16: 34 DG				
**12**	?: 36 DPO	56–59%			19: 41 DG	36–58 DPO		SES 4	1
**13**	?: 49 DPO	77–81%	9: 30–39	46–71%	15: 33 DG	50 DPO		SES 5	2
**14**	?: 30 DPO	47–49%			14: 29 DG	36 DPO		SES 3	1
**15**	?: 41 DPO	63–67%	8: 24–29 DPO	37–52%	17: 35 DG	36–58 DPO		SES 4	1
**16**	?: 24 DPO	38–39%	8: 24–29 DPO	37–52%	14: 29 DG	35b DPO	1: DPO1-2	SES 3	1
**17**	?: 36 DPO	56–59%	8: 24–29 DPO	37–52%	14: 29 DG	35b DPO	2: DPO 13	SES 3	1
**18**	?: 36 DPO	56–59%	8: 24–29 DPO	37–52%	15: 33 DG	35b DPO	2: DPO 13	SES 3	1
**19**	?: 36 DPO	56–59%	8: 24–29 DPO	37–52%	15: 33 DG	35b DPO	2: DPO 13	SES 3	1
**20**	?: 48 DPO	75–79%			17: 35 DG	35b DPO	3: DPO 21		1
**21**	?: 59 DPO	92–97%				36–58 DPO		SES 4	
**22**	?: 36 DPO	56–59%			15: 33 DG	35b DPO	3: DPO 21	SES 4	1
**23**			8: 24–29 DPO	37–52%		58 DPO			
**24**	?: 30 DPO	47–49%			14: 29 DG	36–58 DPO		SES 3	2
**25**	?: 36 DPO	56–59%			15: 33 DG	36–58 DPO		SES 4	1
**26**	?: 30 DPO	47–49%	8: 24–29 DPO	37–52%	17: 35 DG	36–58 DPO		SES 4	
**27**	?: 30 DPO	47–49%				36–58 DPO	2: DPO 13	SES 3	1
**28**	?: 52 DPO	81–85%			19: 41 DG	58 DPO	3: DPO 21	SES 4	3
**29**	?: 41 DPO	63–67%			19: 41 DG	36–58 DPO		SES 4	2
**30**	?: 59 DPO	92–97%	9: 30–39 DPO	46–71%	19: 41 DG	58 DPO		SES 4	3
**31**			8: 24–29 DPO	37–52%		58 DPO			4+
**32**									4+
**33**						50 DPO		SES 6	3
**34**	61–64 DPO		55–65 DPO		68–75 days	~65 DPO		240–300 days	no times or stages available. prf in figures in stage 1, but text says it appears in stage 2
	DPO from another source		NB no stages examined 6–7		ovoviparous	little detail on timing		in total ovoviviparous	


Abbreviations: DPO, days post-oviposition; DG, days of growth. *Nerodia taxispilota* is live bearing and Franklin [30] removed embryos directly from the female during incubation at staged intervals but without an indication of time post fertilisation.

### A. Associated soft tissues of the head

Although our study focuses on skull development in the Egyptian Cobra, associated soft tissue structures are briefly described where they are clearly visible in the cleared and stained specimens, and where, like the venom glands, they contribute to changes in overall head shape. We acknowledge that histological analysis would be needed for a more detailed description of the precise pattern and timing of development of glandular tissues.

#### A1. Venom gland, supra- and infralabial glands

The venom gland is first clearly visible in our 5:17dpo embryo as a thin mass extending along the upper labium from just anterior to the eye to mid-way along the postorbital region (Fig 2A and 2B). At 6a:22dpo, the posterior part has expanded (Fig 3A), and by 6b:24dpo the swollen venom gland has a clear, ramifying internal structure Fig 4A). In the 7a:33dpo embryo, the gland stretches from the otic capsule to the maxilla which it seems to envelope, reaching the anterior margin of the eye, level with the developing fangs (Fig 5A, 5D and 5F). It continues to increase in size and complexity and at 9:47dpo it bulges laterally, covering much of the maxilla (Fig 7E).

**Fig 9:**
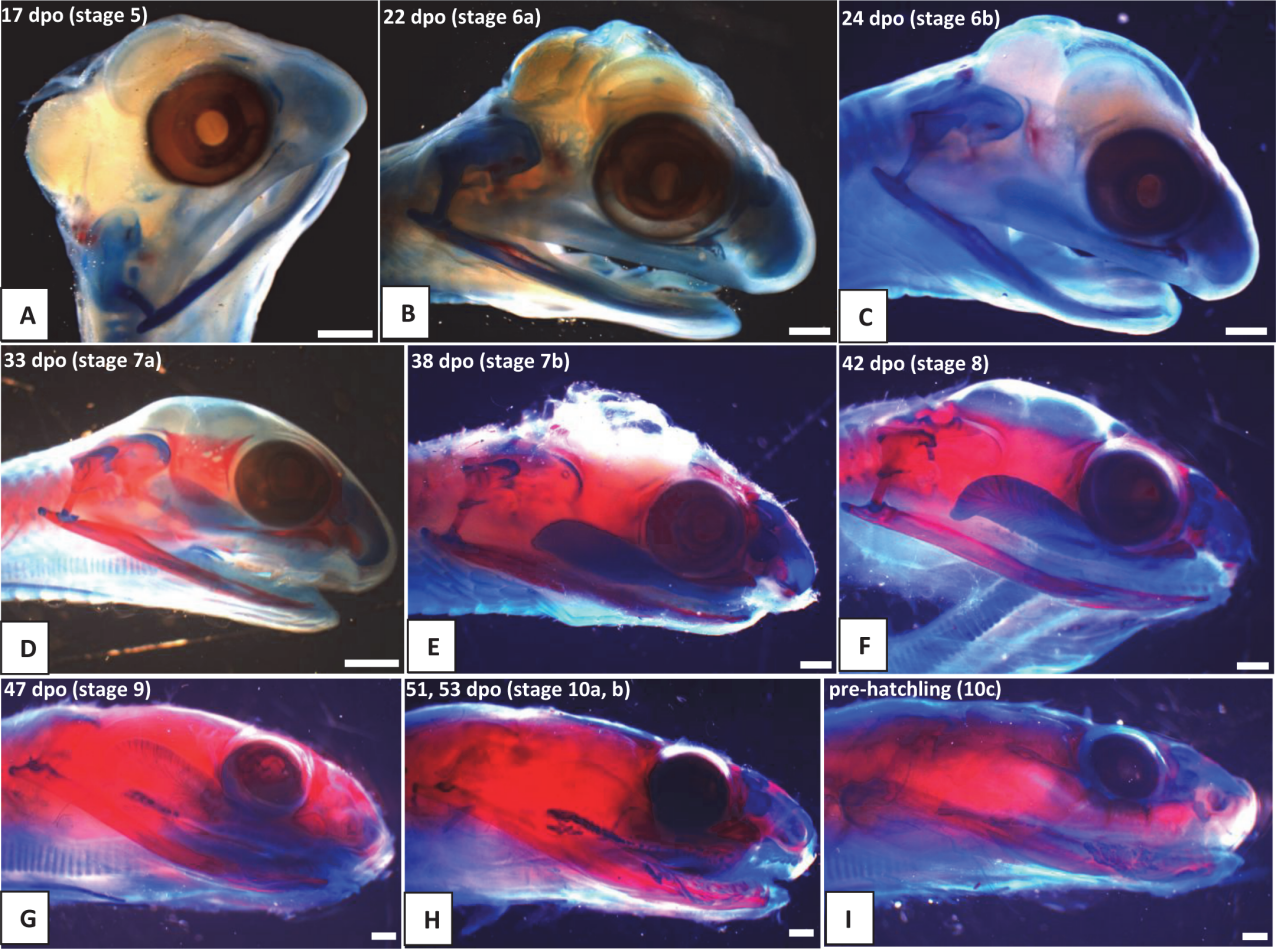
Craniofacial development of *Naja h*. *haje*, changes in head proportions and the venom gland through development. A) 17 dpo; B) 22 dpo; C) 24 dpo; D) 33 dpo; E) 38 dpo; F) 42 dpo; G) 47 dpo; H) 53 dpo; I) Pre-hatching. Scale bars: 1mm.

Accessory mucous salivary glands are present in the upper and lower jaws. The infralabial glands are visible from at least 6a:22dpo, becoming more obvious in later stages. The supralabial glands are most clearly seen in the 10b:53dpo, where individual glands open through a series of ducts (Fig 8B). Fig 9 summarizes the development of the venom gland through different stages.

#### A2. Tongue

The tongue is visible in the 4:13dpo embryo but is small, undivided, and fully tethered to the posterior part of the buccal floor. At 5:17dpo and 6a:22dpo (Fig 3A), the tongue is still short, but the anterior part is more developed and free of the buccal floor and is weakly bifid. It is fully bifid at the 6b:24dpo stage and at 7b:38dpo, the tongue has elongated but remains fully within the mouth. However, by the 9:47dpo stage, the tines of the tongue project out of the mouth onto the upper lip, passing through small grooves on either side of the midline (Fig 7F).

#### A3. Miscellaneous

*Parietal/pineal region*: This is visible only in the 4:13dpo embryo as a deep blue oval on the surface of the developing brain (Fig 1A). In the 6b:24dpo and 7b:38dpo embryos the same oval region is faintly visible, but does not seem to be pigmented (Fig 4D). The last stage at which it is visible, as a slightly darker oval, is 9:47dpo.

*Endolymphatic sacs and maculae*: The endolymphatic sacs are visible in the 4:13dpo embryo as loose aggregations of blue-stained particles that extend towards the otic region (Fig 1A and 1C). The staining changes to red, as evidence of calcium carbonate accumulation, in the 5:17dpo embryos (Fig 2A, 2C and 2E), and two further areas of red staining, one dorsal and one ventral, become visible within the vestibular chamber of the ear, anterior to the stapes. Based on the position of these structures they probably correspond to the saccular and utricular maculae. These structures remain visible in stages 6 (22dpo, 24dpo) and 7 (33dpo, 38dpo), although removal of the skin in the 7b:38dpo embryo has detached the endolymphatic sacs (Fig 6E). By 9:47dpo, the thickened dura obscures these features.

*Face*: In the 4:13dpo embryo, the frontonasal and maxillary prominences have not fused, leaving the lower rostral margin notched (Fig 1B). By 5:17dpo, the prominences are fused (or at least are fully abutting) and the external nares are fully enclosed. The mandibular process is still shorter than the maxillary one, but it does extend anterior to eye (Fig 2A).

*Head shape* (Fig 9): There is a distinct change in head shape between 47 and 51dpo (Fig 9). At 9:47dpo, the profile is lizard-like, but at 10b:53dpo, the profile is more snake-like. The antorbital region is smaller and narrower, the postorbital region has become broader, and the eyes are more prominent than in the previous stage.

### B. Hyoid apparatus

This is visible in the 4:13dpo embryo and is confined to the ventral portion of the head. However, in the 5:17dpo embryo, it is a forked structure, with parallel cornua of roughly 10 vertebral segments in length, and a small anterior lingual process (Fig 2D). The only obvious major change that occurs through subsequent development is elongation of the cornua. At 6a:22dpo (Fig 3B), they are 15 segments in length, and in the 6b:24 dpo embryo (Fig 4C), they extend the length of the preserved specimen, but the body has been cut. In the near-hatchlings, there is some red-staining in the cornua suggestive of calcification or ossification.

### C. Osteocranium

#### C1. Maxilla, premaxilla, and septomaxilla

There is no trace of bone staining in either the 4:13dpo or 5:17dpo embryos, but a dense mesenchymal condensation is present in the position of the maxilla in the latter (Fig 2A). At 6a:22dpo ossification is beginning in the facial process of the maxilla (Fig 3A and 3B), and in the 6b:24dpo embryo, this has extended into a narrow band of ossification in the premaxilla. By stage 7a:33dpo, the maxillary margins are more clearly defined and the triradiate premaxilla is supported by nasal cartilages (Fig 5A, 5D and 5H). There are two large first generation teeth (fangs), although these are not implanted. The very small crowns of the second generation tooth series are also visible, medial to the first. At 7b:38dpo, the low maxillary facial process is overlapped below the eye by the venom gland. The two anterior fangs are prominent, and the second generation replacements lie adjacent to them. The septomaxilla also appears ossified (Fig 6A and 6C). At stage 8:42 dpo, the large maxillary fangs are more strongly recurved (Fig 7A) and by 9:47 dpo, they are attached to the alveolar margin.

#### C2. Circumorbital series

Dorsal mesenchymal condensations are visible in the positions of the prefrontals in the 5:17dpo embryo. Between them and the maxillary condensations is a second ventral condensation. This lies in the position of a lacrimal but is ultimately incorporated into the prefrontal (Fig 2A and 2B). The 6a:22dpo embryo has traces of ossification in the dorsal prefrontal. The ventral condensation is still separate and there is a slender splint-like postorbital condensation behind the eye (Fig 3A). Ossification has progressed by stage 6b:24dpo. The dorsal and ventral prefrontal condensations each contain a centre of ossification separated by a small unossified gap, and the triangular postorbital is now weakly ossified (Fig 4A). In the 7a:33dpo embryo, the two prefrontal centres have merged, but the resulting element remains slender and confined to the orbital rim (Fig 5A and 5B). In subsequent stages, both prefrontal and postorbital remain slender, but they increase their dorsal contacts (Figs. 6C, 6E and 7G). The dorsal edges of the prefrontals extend toward the midline, approaching it at stage 10a:51dpo (Fig 8A and 8C). At 'hatching' (10c:54dpo), the medial part of the prefrontal has aligned itself with the anterior edge of the frontal (Fig 8E). At 9:47dpo, the postfrontal meets the parietal (weakly ossified), and by stage 10a:51dpo has contacted, or nearly contacted, the frontal to exclude the parietal from the orbital rim.

#### C3. Roofing bones (nasal, frontal, parietal)

In the 5:17dpo embryo, mesenchymal condensations are just visible in the orbital margin of the frontal, the ventrolateral flange of the parietal and the mid-region of the nasal (Fig 2A and 2B). The 6a:22dpo embryo shows a radiating pattern of condensation (or blood vessels) within the ventrolateral flange of parietal (Fig 3A), followed at stage 6b:24 dpo by ossification in the same region and in the preorbital edge of the frontal (Fig 4A and 4B). In the 7a:33 dpo embryo, the frontal ossification has extended along the orbital margin and into anteroventral flanges that extend down to contact the unossified trabeculae cranii (which reach the nasal capsules anteriorly) but not the narrow midline parasphenoid rostrum. The triangular dorsal plates of the nasals are also ossified, as are the medial flanges that will contribute to the prokinetic joint with the frontals (Fig 5A). Each of the roofing bones has enlarged in the 7b:38dpo embryo and the nasals meet the frontals in the midline, although nasal cartilages separate the nasals and frontals for most of their width. These cartilages also support the small ventral premaxilla (Fig 6A and 6C). The frontals have almost reached the dorsal and ventral midlines, but they do not make contact with the parasphenoid rostrum until stage 8:42dpo. At this stage, the parietals are still limited to the lateral surfaces of the head and do not roof the brain, but they extend posteromedially around the dorsal skull margin towards the endolymphatic sacs (Fig 7C). By stage 9:47dpo, thin posterolateral parietal laminae cover the margins of the brain, but the median region is still open, and this large central fontanelle persists through to the 10b:53dpo embryo (Fig 8C). In the near hatching embryos (10c:54dpo), the fontanelle seems to have closed in one specimen, but it remains open in a second individual (Fig 8E).

#### C4. Palate

The first trace of ossification in the palate is seen in the 6a:22dpo embryo, with a weak centre in the ectopterygoid and threads of red stain in the palatine and pterygoid (Fig 3B). This is followed in the 6b:24dpo embryo by traces in the vomer. The extent of ossification in each palatal element (as shown by alizarin staining) increases at stage 7 (33dpo, 38dpo) and the ectopterygoid makes contact with the posterior tip of the maxilla (Fig 5D). The palatal bones appear to be almost fully ossified at 8:42dpo, but they are slender and the pterygoid ends in a tapered tip anterior to the quadrate. Small teeth are visible in the tissues below the palatine and pterygoid but they do not appear to become attached until stage 10b:53dpo.

#### C5. Quadrate and suspensorium

A short, almost vertical quadrate cartilage is visible in the 4:13dpo embryo. It meets Meckel's cartilage in the lower jaw, but the joint between them does not seem to have formed (Fig 1A). At 5:17dpo, the cartilage is longer but still almost columnar with only a slight broadening of the dorsal end. A thin unstained (alcian blue) region between them suggests a joint cavity is now present (Fig 2A and 2C). The 6a:22dpo embryo has a quadrate that is closer to the adult shape. There is no trace of ossification, but the proximal end is expanded anteriorly. Dorsal to it, wedged between quadrate and otic capsule, is a slender splint-like ossification which is the first rudiment of the supratemporal (Fig 3A and 3D). By stage 6b:24dpo, there is some perichondral ossification in the quadrate and the supratemporal has expanded anteriorly (Fig 4B). Ossification progresses in both elements and by stage 7b:38dpo, the quadrate is almost fully ossified except at its dorsal and ventral extremities and the supratemporal is close to the adult shape, although it does not reach anterior to the prootic (Fig 6A and 6E). At stage 10a:51dpo, with the widening of the posterior skull, the quadrate becomes more oblique (in coronal view), angling from dorsomedial to ventrolateral (Fig 8C).

#### C6. Otic capsule and stapes

The otic capsule is visible, but only weakly alcian blue stained, in the youngest (4:13dpo) embryo (Fig 1A and 1C). It is more distinct at 5:17dpo and a cartilage stapes is already visible (Fig 2A and 2C). Seen in dorsal view (Fig 2E), the medial corner of the otic capsule (containing the common crus, the junction of anterior and posterior semicircular canals) is visible on each side. However, these corners are separated by a gap in which the rest of the supraoccipital will ultimately form. There is also strong alcian blue staining ventrally in the region of the basal plate. The three semicircular canals are very well defined in the 6a:22dpo embryo (Fig 3C), but there seems to be little development of the surrounding otic capsule itself. In the posterior midline, between the otic capsules, the paired dorsal laminae of the occipital arches are large and triangular. These laminae have fused in the 6b:24dpo embryo (Fig 4C) to form a median plate which, for this description, we will simply call the intercapsular plate (as its homologies are controversial, see Discussion). Anterolaterally, the intercapsular plate fuses with the anteromedial corner of each otic capsule, but plate and capsule remain separated posterolaterally by a cleft (Fig 4D). This is the occipitocranial fissure [18], the dorsal part of the metotic fissure.

In the 7a:33dpo embryo, the intercapsular plate remains unossified but ossification has spread from the basal plate into the lower parts of the occipital arches, where the exoccipitals will form (Fig 5C). The ossification does not extend into the roof of the foramen magnum, but there seems to be a little posterior process on the exoccipital on each side. Although there is no ossification in the intercapsular plate, there is an ossification centre in the anteromedial corner of the otic capsule on each side, at the junction of anterior and posterior semicircular canals (Fig 5C). These correspond in position to the epiotic centres described by Parker [19] as they are quite distinct from any other ossification centre in or between the otic capsules. Ossification has spread through the otic capsule at this stage, but has not extended over the anterior or posterior semicircular canals, except at that discrete anteromedial corner. The ossification in the otic capsule is strongest anteroventrally, anterior to the fenestra vestibuli. The capsule immediately ventral to the fenestra vestibuli is not yet ossified, but there is a strong band of ossification across the posterior, basioccipital, region of the basal plate.

In the 7b:38dpo embryo (Fig 6E), the otic capsules are almost fully ossified except for the anterior rim of the anterior semicircular canal, the sutures between components, and between the capsules in the region of the intercapsular (future supraoccipital). The epiotic component of the supraoccipital is ossified, and ossification has spread from this into the intercapsular plate immediately adjacent to it. A suture (blue) separates the epiotic component from the rest of the otic capsule which is ossifying as prootic and opisthotic. The anteromedial part of the supraoccipital is still cartilage. Posterolaterally, the intercapsular plate remains separated from the otic capsule by the occipitocranial fissure. The exoccipital ossification has extended further dorsally, but the dorsal margin of the foramen magnum is still cartilage. The stapes is ossified medially and its footplate sits in a simple fenestra vestibuli, the ventral margin of which is still cartilaginous. The prootic notch appears to lack a laterosphenoid.

**Fig 10:**
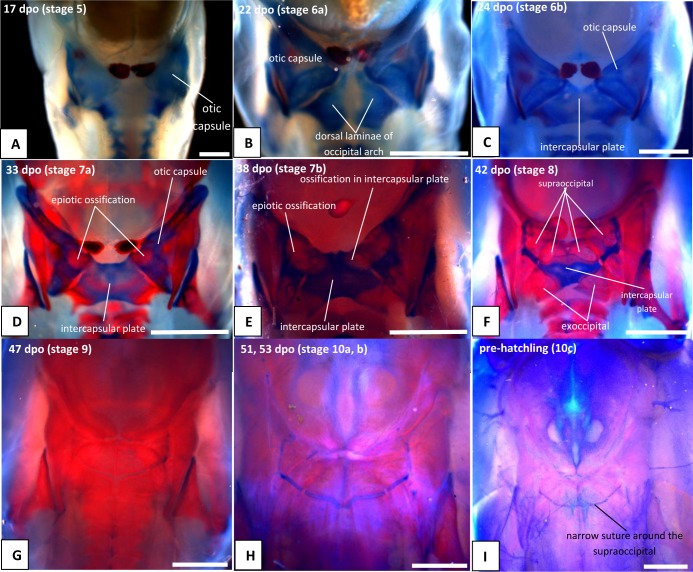
Craniofacial development of *Naja h*. *haje*, development of the occipital region. A) 17 dpo; B) 22 dpo; C) 24 dpo; D) 33 dpo; E) 38 dpo; F) 42 dpo; G) 47 dpo; H) 53 dpo; I) Pre-hatching. Note that the red bar that appears to run horizontally across the developing supraoccipital in D is actually an ossification in the basal plate further ventrally. Scale bars: 1mm.

The otic capsule at 8:42dpo (Fig 7C and 7D) is fully ossified except in the suture between it and the supraoccipital, and between prootic and opisthotic. The posterior part of the intercapsular plate is still cartilage and only just enters the foramen magnum due to lack of ossification in the exoccipital/ otoccipital. The capsule now fits against a recess in the side of the parietal anteriorly (Fig 7D). There is a laterosphenoid dividing the prootic foramen and a crista circumfenestralis formed by an extra flange on the opisthotic. At 9:47dpo (Fig 7G), sutures remain between the narrow supraoccipital (completely excluded from the foramen magnum) and the rest of the capsule. The exoccipitals meet in the midline behind the supraoccipital. This is relatively unchanged at 10a:51dpo (Fig 8C), except that the sutures between the supraoccipital and the rest of the otic capsule are narrower. In the prehatchling embryos, one specimen has a separate centre of ossification in the intercapsular region between the medial edges of the exoccipitals. A summary of the development and changes in the otic capsule and stapes is shown in Fig 10.

#### C7. Chondrocranium and basicranium

The chondrocranium of N. h. haje has been described by El Toubi et al. [20] and it is only covered here where it is relevant to osteocranial structures.

The trabeculae cranii are visible in the 5:17dpo and 6a:22dpo embryos, extending much of the length of the roof of the mouth. They run in parallel anteriorly, then diverge posteriorly where they meet the acrochordal cartilage (sensu Rieppel and Zaher [18], future crista sellaris), forming a triangular pituitary fenestra. The narrow acrochordal cartilage separates the pituitary fenestra from a larger wider basicranial fenestra. The trabeculae remain paired anteriorly, but as development progresses, the parallel anterior parts of the trabeculae are separated by a slender parasphenoid rostrum and then, gradually, bone invades their posterior ends from the dorsum sellae. In the 6b:24dpo embryo, there is ossification into the posterior (basioccipital) part of the basal plate and into the ventral parts of the occipital arches (exoccipitals).

The basicranium of the 7a:33dpo embryo (Fig 5B and 5C) retains large basicranial and pituitary fenestrae but there is ossification into their margins, including the acrochordal cartilage, forming the crista sellaris. Anteriorly, the trabeculae meet the large nasal capsule, and a narrow parasphenoid rostrum is visible between them in the anterior braincase floor, although it does not yet connect to the basicranium. There is an ossification across the position of the crista sellaris and into the bases of the trabeculae cranii but not along their full length. The posteriormost part of the basal plate is strongly ossified, with ossification beginning to spread anteroventrally to the region below the basicranial fenestra. The stapes is ossified and the footplate sits in a simple fenestra vestibulae (i.e. no evidence of the crista circumfenestralis). By stage 7b:38dpo, the parasphenoid rostrum has made contact with the ossifying basisphenoid but the trabeculae are still clearly visible and meet basisphenoid anterolaterally. By stage 8:42dpo, there is thin bone flooring the pituitary and basicranial fenestrae, and paired foramina in the pituitary fenestra region (Fig 7C). The basicranial region is not visible in later stages because of the underlying jaws and the buccal floor.

#### C8. Lower jaw and articulations

The core structure of the lower jaw, Meckel's cartilage, is visible in the 4:13dpo embryo (Fig 1A) but it is still shorter than the upper jaw. The joint between it and the quadrate is unclear and may be at an early stage of formation. At stage 5:17dpo Meckel's cartilage is longer and the anterior tip bears the distinctive inward curvature seen in later embryos (Fig 2D). There is a clearer separation between quadrate and articular regions, and there is a distinct retroarticular process (Fig 2C). The first traces of ossification are seen in the 6a:22 dpo embryo (Fig 3A, 3B and 3D), with discontinuous slivers of bone in the region of the prearticular, surangular, and dentary. The jaw joint is more distinct, and there is a denser lip of cartilage in front of the quadrate, a smaller lip behind it, and some shaping of the retroarticular process (Fig 3D). Meckel's cartilage remains visible as a continuous structure from the anterior tip into the cartilaginous articular region. By stage 6b:24dpo, the entire mandible has a thin sheath of bone (Fig 4A) but Meckel's cartilage remains patent throughout. By 7a:33dpo, the sheath of bone around the mandible has clearly differentiated into the compound bone and dentary (Fig 5D). The angular and splenial are also visible as thin sheets (Fig 5D). Ventrally, the intramandibular joint is demarcated by small 'lips' on either side of it, although Meckel’s cartilage persists through it without any apparent change in diameter (Fig 5E). Ossification has extended into the anterior part of the articular, but the retroarticular process and the area around the quadrate-articular joint remain cartilaginous (Fig 5F). Dentary teeth appear for the first time in the 7b:38dpo embryo, as a line of very small, unattached, tooth crowns. Meckel's cartilage remains visible (Fig 6D), but is partly ossified in its posterior half within the compound bone, so that the cartilaginous section extends only just beyond the intramandibular joint. Anterior to each dentary, the inwardly curving tip of Meckel's cartilage extends toward the midline but the bilateral tips do not meet (Fig 6D).

The 8:42dpo shows relatively little change, except that there are at least two generations of small unattached teeth on the dentary (Fig 7A). By stage 9:47dpo, only the tip of the retroarticular process remains cartilaginous, but Meckel's cartilage is still weakly visible through the core of the mandible up to, and slightly beyond, the intramandibular joint. There are at least three generations of dentary teeth (Fig 7E and 7F), but the attachment of the first tooth generation does not occur until stage 10b:53dpo (Fig 8A), and then only anteriorly. This generation is fully attached, all along the dentary, only in the prehatchling individuals.

## Discussion

### Comparison with Kamal et al. [12]

Kamal et al. [12] gave a detailed account of the osteocranium in both the embryonic and adult cobra, *Naja h*. *haje*. However, they had only nine eggs collected from a 'wild' nest. Two were opened on the day of collection and the authors recorded that the embryos were 'nearly at middle of their embryonic life'. These they designated Group 1. The remaining seven eggs were incubated 'at room temperature'. Two more eggs were opened seven days after collection (Group 2), a further two 17 days after collection (Group 3), and two more 27 days after collection (group 4). The last egg hatched at 31 days after collection. From our own observations, room temperature in Cairo during the breeding period of *N*. *h*. *haje* would typically range between 32–35° during the day, but could be substantially cooler at night. Of course, diurnal temperature fluctuations also occur In the wild, but the female snake can influence this by choice of breeding site (e.g. depth, cover). Both incubation temperature generally and fluctuations in temperatures are known to affect embryo development (e.g., [16,21]). Without knowing the dpo of Kamal et al.'s eggs when first recovered, nor details of the incubation regime, precise comparison is difficult. However, for our material, we recorded an incubation period of 51–55 days at 30°C. On that basis, Kamal et al.'s youngest embryos (31 days prior to hatching) could have been about 21–24 dpo (our Stage 6), and their Group 3 individual (used for the description of the embryo skull) about 41 dpo (our Stage 8). Although this is a rough estimate given the uncertainties, it is reasonably consistent with the described morphology of their Group 3 specimen, which is intermediate between our 7b:38dpo and 8:42dpo embryos in terms of skull development.

**Age estimate based on K&E hatching:**

- Gp 1: X days (total body length 98.2 mm): 24 days (6b)
- Gp 2: X+7 days (TBL 133.4 mm): 31 days (7a)
- Gp 3: X+17 days (TBL 166 mm): 41 days (8a)
- Gp 4: X+27 days (TBL 197 mm): 51 days (10a)
- Gp 5: X+31 (hatch): ~55 days (10b)

K&E average length at that age:

- Gp 1: X days (total body length 98.2 mm): 130mm
- Gp 2: X+7 days (TBL 133.4 mm): 211mm
- Gp 3: X+17 days (TBL 166 mm): 250mm
- Gp 4: X+27 days (TBL 197 mm): 260mm
- Gp 5: X+31 (hatch): 300mm

Khannoon and Evans [15] recorded total body lengths for near hatchlings at 200–400 mm. At 197 mm total length, Kamal et al's [12] Group 4 embryo is at the extreme lower end of that scale. However, this again could be an incubation temperature effect (e.g. [16,21,22]).

Kamal et al. [12] do not figure the skull of their Group 1 and Group 2 embryos, but they do record some features. Many of these agree with our observations of embryos estimated to be about the same age, but there are some points of difference. They found that the maxilla, palatine and pterygoid of their Group 1 embryos (~24dpo) were more strongly ossified than other elements, and therefore concluded that the primordia of these bones had arisen first. In fact, the primordia of the frontals, parietals, prefrontals and maxillae were present, but not ossified, in our 5:17dpo embryos. At 6a:22dpo, the first clear ossification centres are in the supratemporal, prearticular and surangular, with very weak ossification visible in parts of the maxilla, prefrontal, and dentary. In the palate, the ectopterygoid is more heavily stained than either the palatine or pterygoid which appear as thin threads. Kamal et al. [12] also described the frontals of their Group 1 embryo as arising over the posterior part of the nasals, whereas in our specimens they are first visible along the orbital margins (as in most other squamates, [1,10,23]).

Kamal et al. [12] regarded the single bone posterior to the orbit as a postfrontal, but from the first appearance of its primordium at 6a:22dpo (as a thin streak), it is never in contact with either the frontal or parietal, nor is there any indication of two centres that fuse. Thus the bone is in the position of the postorbital of all limbed squamates and we follow Cundall and Irish (2008) in regarding it as such in snakes, including *Naja*.

There are small differences between their figured Group 3 embryo and our specimen at this stage, but there is generally nothing significant. However, our more complete series has allowed us to clarify the ossification sequence for this species, to estimate the age of their embryonic specimens, and to provide a more detailed basis for the comparison of *N*. *h*. *haje* with other snakes. Moreover, our observations of the development of the supraoccipital and exoccipital differ strikingly from theirs, and also from that of other authors, as discussed below.

### The development of the supraoccipital

Classical accounts (e.g. [1]) reported that two different embryological components can contribute to the posterior roof of the braincase. The first is designated tectum synoticum and, as its name suggests, it extends between the two otic capsules and is derived solely from capsular material. The second is the tectum posterius, which is derived from the dorsal parts of the occipital arches. These, in turn, are derived from the neural arches of cranial vertebrae [18] and are therefore post-otic somitic derivatives. Both de Beer [1] and Bellairs and Kamal [6] related these embryological components to two different supraoccipital types: one is formed solely by the tectum synoticum and the other formed by a combination of the tectum synoticum and the tectum posterius (= occipital tectum). According to both De Beer [1] and Bellairs and Kamal [6], snakes belong to the first of these groups and lack a tectum posterius. De Beer [1] based his conclusion on '*Tropidonotus natrix'* (= *Natrix natrix*), saying that in this snake 'the occipital arches fail to meet but just join the tectum synoticum, and there is apparently no tectum posterius'. Bellairs and Kamal [6], summarising work by Kamal and Hammouda [7], stated that whereas a stage 5:29dpo embryo of *Psammophis* lacked any connection between the otic capsules, a stage 6:35dpo embryo had a rudimentary tectum synoticum of otic origin. In the same embryo, the occipital arches were said to be separated in the dorsal midline. A stage 7:44dpo embryo reportedly had a complete tectum synoticum roofing the foramen magnum. Rieppel and Zaher [18], describing the development of the snake *Acrochordus*, reached the same conclusion, although they noted that the distinction between the tectum synoticum and tectum posterius was not always clear.

We cannot comment on the interpretations outlined above without reference to a comparable series of embryos for the relevant snakes. However, our embryo series of the Egyptian cobra shows the stages in the development of the posterior cranial roof very clearly (Fig 10). The occipital arches developed large dorsal laminae (Fig 10B) that first fused medially to one another, and then fused to the anteromedial margins of the otic capsules (Fig 10C). In the description above, we have deliberately used a neutral term, intercapsular plate, for this median cartilage roof but as it is unequivocally derived from the occipital arch, it corresponds to the tectum posterius of De Beer [1] (p245,1985 reprint). It completely fills the space between the otic capsules anteriorly. There is no separate cartilaginous tectum synoticum.

There are two possibilities for this disparity. The first is that some earlier researchers had too few stages to determine the homologies of the intercapsular tectum accurately, and therefore misidentified the component parts. The second is that there is genuinely variation in the way the intercapsular tectum develops. El Toubi and Kamal [24] provided some support for the latter conclusion. They recorded that whereas *Malopolon*, *Natrix*, *Psammophis* and *Thelotornis* appeared to have a tectum derived from the otic capsules (tectum synoticum), the tectum in *Lamprophis*, *Crotaphopeltis* and *Dasypeltis* appeared to have formed primarily from the occipital arches. El Toubi et al. [25] give a complementary list, with *Vipera aspis* and *Vipera russeli* recorded as having a tectum formed mainly or exclusively from the occipital arches, but *Cerastes* and *Eryx* as having a purely capsular tectum. If this difference really exists, and is not simply a matter of interpretation, then it is of interest and needs further work in squamates generally, because the embryonic origins of the tectal components appear fundamentally different. In their review of the chondrocranium, Bellairs and Kamal [6] reported that most lizards have a tectum formed from both tectum synoticum and tectum posterius, but that the condition in some gekkotans (e.g. *Ptyodactylus*) is uncertain. De Beer [1] recorded *Sphenodon* as lacking a tectum posterius.

A second point of controversy relates to ossification centres in the developing supraoccipital. Parker [19] described the supraoccipital as developing from distinct epiotic ossification centres that formed at the dorsomedial edges of the otic capsules. Subsequent workers (e.g. [1,6]) either doubted or denied the existence of these centres, particularly in snakes. De Beer [1] described the supraoccipital in *'T*. *natrix'* as developing in the anterior and lateral parts of the tectum synoticum and extending into the roof of the auditory capsule on each side, that is from medial to lateral. He also reported that *Vipera aspis* and '*Leptodeira' hotamboia* (= *Crotaphopeltis*) showed the same pattern. In relation to epiotic ossifications, he wrote (p.251, 1985 reprint) 'These lateral portions of the supraoccipital were regarded by Parker as epiotics but his account of their independent ossification has not been confirmed'. Similarly, in their description of *N*. *h*. *haje*, Kamal et al. [12] wrote: 'The supraoccipital arises from a pair of perichondral lamellae which are formed on the dorsal and ventral surfaces of the anterior part of the tectum synoticum. Laterally, on either side, the supraoccipital extends slightly in the roof of the auditory capsule. This lateral part of the supraoccipital cannot be considered as an epiotic bone since its independent ossification is not observed. Thus the two epiotic bones are considered to be completely lacking in *Naja*.' Again, we cannot comment on other snakes, as we do not have developmental series to hand, but for *N*. *h*. *haje*, the embryos show separate epiotic ossification centres to be present (Fig 10D and 10E). They are distinct from the rest of the otic capsule which ossifies separately. Moreover, ossification spreads lateral to medial, from these epiotic centres into the intercapsular plate that subsequently makes up the body of the supraoccipital. The two ossification fronts meet to form a single bone unit around stage 8:42dpo. Again, further work is needed to establish whether or not there is genuine variation in the way the supraoccipital ossifies within squamates and, if so, at what level this variation occurs (i.e., intra- or interspecifically, or at larger phylogenetic scales).

### Comparison with *Naja kaouthia*

Jackson [8] described both external and skeletal stages of the Asian Monacled Cobra, *N*. *kaouthia*. In our comparison of external features, we found minor differences in growth patterns and more obvious ones in the appearance of head scales, but this may have reflected differences in incubation temperature that are known to affect development (e.g., [22]).

In comparing her skull development stages with those of *N*. *h*. *haje* (summarised in Tables 1 and 2), we found a general similarity for most developmental events, but Jackson recorded ossification in the dentary, ectopterygoid, palatine, pterygoid, premaxilla, and articular earlier in *N*. *kaouthia* than in *N*. *h*. *haje*, in terms of both stage (3 versus 6–7a) and dpo (9–15 versus 22–33). In the 4:13 dpo embryo of *N*. *h*. *haje*, there is no trace of the skull elements, even as mesenchymal condensations. However, these observations may not be fully comparable. Jackson noted that although her stage 3 embryo had some bone, this did not stain with alizarin. The maxilla, ectopterygoid, and pterygoid were described as being visible as grey translucent bones, with the dentary and articular beginning to ossify in the lower jaw. This is more comparable to our Stage 6 (22 dpo) embryo, although unstained mesenchymal condensations are visible in the 5:17dpo embryo in the region of the future maxilla, prefrontal, frontal and parietal. Given the rather small difference between Jackson's figures for Stage 3 and Stage 7, compared to the significant difference between Stages 7 and 8, there may be an error. Jackson's Stage 7 (24–28dpo) is quite similar to our late stage 6 embryo of similar age (24dpo), as is her Stage 8 embryo (28–38dpo) compared to our Stage 7 (33 dpo embryo), again highlighting the inconsistency for the Stage 3 result. At Stage 10 (hatchling), *N*. *kaouthia* and *N*. *h*. *haje* have reached a comparable level of development, except that Jackson reported the frontals and parietals had yet to meet in the midline. The frontals do meet in both hatchlings of *N*. *h*. *haje*, and the parietals come very close to doing so.

### Comparison with other snakes

Tables 1 and 2 provide a summary of the ossification sequence in *Naje h*. *haje*, compared to published descriptions of development in other snakes. In each case, we have given the stage, the age in days post oviposition (DPO) and, where possible, the appearance in terms of percentage development time. Detailed comparison is limited by differences in reporting, staging method, and incubation temperature. There is a general similarity in ossification sequence, but the early appearance of several skull bones in *Python sebae* [9], compared to other snakes, is striking. It is consistent, however, with the very early appearance of calcified endolymphatic sacs in that taxon [9,15]. In snakes, as in lizards, most of the calcium for early skeletogenesis comes from the yolk (e.g., [26, 27]), although this can be supplemented closer to hatching by mobilization of calcium from the eggshell. Differences in the initial levels of calcium within the yolk appear to be correlated with hatchling bone density (Tables 1 and 2 in [28]) but, potentially, the speed and mode of calcium mobilization from the yolk could affect the timing of onset of ossification. However, this is a area on which little is published.

## Conclusions

The availability of a detailed developmental series for the Egyptian cobra, *N*. *h*. *haje*, provides a more comprehensive basis for comparison with other snakes. It has allowed us to supplement and extend the work of Kamal and colleagues [7,12–14] by providing data on early embryonic stages that were not available to them and by providing a full set of colour images. As a result, some errors in the suggested sequence of ossification have been corrected. Most importantly, the new data has re-opened the debate with respect to supraoccipital development in snakes and other reptiles, and should prompt further work in this area.

## References

[1] De Beer GR. The development of the vertebrate skull Reprinted 1985th ed. Chicago: University of Chicago Press; 1937.

[2] Carroll RL. Vertebrate paleontology and evolution New York: W.H. Freeman and Company; 1988.

[3] Cundall D, Irish F. The snake skull In: Gans C, Gaunt AS, Adler K, editors. Biology of the Reptilia, Volume 20, Morphology H. New York: Society for the Study of Reptiles and Amphibians; 2008 pp. 349–692.

[4] Hofstadler-Deiques C. The development of the pit organ of *Bothrops jararaca* and *Crotalus durissus terrificus* (Serpentes,Viperidae): support for the monophyly of the subfamily Crotalinae. Acta Zool. 2002;83: 175–182.

[5] Hofstadler-Deiques C, Walter M, Mierlo F, Ruduit R. Software system for three-dimensional volumetric reconstruction of histological sections: a case study for snake chondrocranium. The Anat Rec. 2005;286A: 938–944. 10.1002/ar.a.20227 16114067

[6] Bellairs A d'A, Kamal AM. The chondrocranium and development of the skull in recent reptiles In: Gans C, Parsons TS, editors. Biology of the Reptilia, Volume 11, Morphology F. New York: Academic Press; 1981 pp. 1–263.

[7] Kamal AM, Hammouda HG. The development of the skull of *Psammophis sibilans* II. The fully formed chondrocranium. J Morphol. 1965;116: 247–296.

[8] Jackson K. Post-ovipositional development of the Monocled cobra, *Naja kaouthia* (Serpentes: Elapidae). Zoology 2002;105: 203–214. 16351869 10.1078/0944-2006-00077

[9] Boughner JC, Buchtova M, Katherine Fu, Diewert V, Hallgrımsson B, Richman JM. Embryonic development of *Python sebae*—I: Staging criteria and macroscopic skeletal morphogenesis of the head and limbs. Zoology 2007;110: 212–230 17499493 10.1016/j.zool.2007.01.005

[10] Boback SM, Dichter EK, Mistry HL. A developmental staging series for the Africa, house snake, *Boaedon* (*Lamprophis*) *fuliginosus* . Zoology 2012;115: 38–46. doi: 10.1016/j.zool.2011.09.001 22206643 10.1016/j.zool.2011.09.001

[11] Polachowski KM, Werneburg I. Late embryos and bony skull development in *Bothropoides jararaca* (Serpentes, Viperidae). Zoology 2013;116: 36–63. doi: 10.1016/j.zool.2012.07.003 23348050 10.1016/j.zool.2012.07.003

[12] Kamal AM, Hammouda HG, Mokhtar FM. The development of the osteocranium of the Egyptian Cobra: I. The embryonic osteocranium. Acta Zool. 1970a: 1–17.

[13] Kamal AM, Hammouda HG, Mokhtar FM. The development of the osteocranium of the Egyptian Cobra: II. The median dorsal bones, bones of the upper jaw, circumorbital series, and occipital ring of the adult osteocranium. Acta Zool. 1970b: 19–30.

[14] Kamal AM, Hammouda HG, Mokhtar FM. The development of the osteocranium of the Egyptian Cobra: III. The otic capsule, palate, temporal bones, lower jaw and hyoid apparatus of the adult osteocranium. Acta Zool. 1970c: 31–42.

[15] Khannoon E, Evans SE. The embryonic development of the Egyptian cobra *Naja h*. *haje* (Squamata: Serpentes: Elapidae). Acta Zool. 2013;95: 472–483.

[16] Deeming DC, Ferguson MWJ. Egg incubation: its effects on embryonic development in birds and reptiles Cambridge: Cambridge University Press; 1991.

[17] Hanken J, Wassersug R. The visible skeleton. A new double-stain technique reveals the nature of the hard tissues. Funct Photog. 1981;16: 22–26.

[18] Rieppel O, Zaher H. The development of the skull in *Acrochordus granulatus* (Schneider) (Reptilia: Serpentes) with special consideration of the otico-occipital complex. J Morphol. 2001;249: 252–266. 11517468 10.1002/jmor.1053

[19] Parker WK. On the structure and development of the skull of the common snake (*Tropidonotus natrix*). Philos T R Soc B. 1879a;169: 385–417.

[20] El-Toubi MR, Kamal AM, Mokhtar FM. The chondrocranium of late embryos of the Egyptian cobra, *Naja haje* . Anat Anz. 1970;127(3): 233–89. 5520286

[21] Booth DT. Influence of incubation temperature on hatchling phenotype in reptiles. Physiol Biochem Zool. 2006;79: 274–281. 16555187 10.1086/499988

[22] Burger J. Effects of incubation temperature on behaviour of young Black Racers (*Coluber constrictor*) and Kingsnakes (*Lampropeltis getulus*). J Herpetol. 1990;24: 158–163. 2396957

[23] Parker WK. On the structure and development of the skull in the Lacertilia. Part I. On the skull of the Common Lizards (*Lacerta agilis*, *L*. *viridis*, and *Zootoca vivipara*). Philos T R Soc B. 1879b;170: 595–640

[24] El Toubi MR, Kamal AEM. The origin of the tectum of the occipitoauditory region in Squamata. Proc Egyptian Acad Sci. 1965;18: 73–75

[25] El Toubi MR, Kamal AM, Zaher MM. The development of the chondrocranium in the snake, *Malpolon monspessulana* . Acta Anat. 1973;85: 593–619 4793087

[26] Packard MJ. Patterns of mobilization and deposition of calcium in embryos of oviparous amniotic vertebrates. Israel J Zool. 1994;40: 481–492.

[27] Stewart JR, Ecay TW, Blackburn DG. Sources and timing of calcium mobilization during embryonic development of the Corn Snake, *Pantherophis guttatus* . Comp Biochem Phys A. 2004;139: 335–341. 10.1016/j.cbpb.2004.09.016 15556389

[28] Stewart JR, Ecay TW. Patterns of maternal provision and embryonic mobilization of calcium in oviparous and viviparous squamate reptiles. Herpetol Conserv Biol. 2010;5: 341–359.

[29] Haluska F, Alberch P. The cranial development of *Elaphe obsoleta* (Ophidia Colubridae). J Morphol. 1983;178: 37–55. 10.1002/jmor.1051780104 30068058

[30] Franklin MA. The embryonic appearance of centres of ossification in the bones of snakes. Copeia 1945: 68–72.

[31] Korneva LG. Embryonic development of the water snake (*Natrix tessellata*). Zool Zh. 1969;98: 110–120.

